# Brain Tumor Classification in MRI Scans Using Edge Computing and a Shallow Attention-Guided CNN

**DOI:** 10.3390/biomedicines13102571

**Published:** 2025-10-21

**Authors:** Niraj Anil Babar, Junayd Lateef, ShahNawaz Syed, Julia Dietlmeier, Noel E. O’Connor, Gregory B. Raupp, Andreas Spanias

**Affiliations:** 1 Sensor Signal and Information Processing (SenSIP) Center, Arizona State University, Arizona, AZ 85281, USA; nbabar@asu.edu (N.A.B.); jlateef@asu.edu (J.L.); sasyed9@asu.edu (S.S.); spanias@asu.edu (A.S.); 2 Insight Research Ireland Centre for Data Analytics, Dublin City University, Glasnevin, D09V209 Dublin, Ireland; noel.oconnor@insight-centre.org; 3 School for Engineering of Matter, Transport and Energy (SEMTE), Arizona State University, Arizona, AZ 85281, USA; raupp@asu.edu

**Keywords:** biomedical image processing, convolutional neural networks, edge computing, image classification, magnetic resonance imaging, model compression

## Abstract

**Background/Objectives:** Brain tumors arise from abnormal, uncontrolled cell growth due to changes in the DNA. Magnetic Resonance Imaging (MRI) is vital for early diagnosis and treatment planning. Artificial intelligence (AI), especially deep learning, has shown strong potential in assisting radiologists with MRI analysis. However, many brain tumor classification models achieve high accuracy at the cost of large model sizes and slow inference, limiting their practicality for medical edge computing. In this work we introduce a new attention-guided classification model and explore how model parameters can be reduced without significantly impacting accuracy. **Methods:** We develop a shallow attention-guided convolutional neural network (ANSA_Ensemble) and evaluate its effectiveness using Monte Carlo simulations, ablation studies, cross-dataset generalization, and Grad-CAM-generated heatmaps. Several state-of-the-art model compression techniques are also applied to improve the efficiency of our classification pipeline. The model is evaluated on three open-source brain tumor datasets. **Results:** The proposed ANSA_Ensemble model achieves a best accuracy of 98.04% and an average accuracy of 96.69 ± 0.64% on the Cheng dataset, 95.16 ± 0.33% on the Bhuvaji dataset, and 95.20 ± 0.40% on the Sherif dataset. **Conclusions:** The performance of the proposed model is comparable to state-of-the-art methods. We find that the best tradeoff between accuracy and speed-up factor is consistently achieved using depthwise separable convolutions. The ablation study confirms the effectiveness of the introduced attention blocks and shows that model accuracy improves as the number of attention blocks increases. Our code is made publicly available.

## 1. Introduction

The brain consists of nerves and connective tissue and is the most complex and vital human organ. As a biological neural network, the brain contains a system of more than 89 billion communicating neurons with the transmission rate of 10 to 100 signals per second [1]. The brain controls most physical tasks, including awareness, movements, breathing, digestion, sensations, thoughts, speech, and memory. The diseases that disrupt brain function have a profound negative impact on people’s lives, their families, and communities. Brain tumors develop when aggressive, rapid, and uncontrollable cell growths happen in the brain. This disease causes alteration to neuronal networks, has the highest mortality rate [2], and is the 10th leading cause of death for adults worldwide [3]. Without medical intervention, this condition can lead to cognitive and motor deficits, visual impairment, speech impairment, paralysis, and often death [4]. There exist more than 100 forms of different brain tumors [5] that can be classified into primary and secondary categories depending on their source. Primary brain tumors originate in the primary brain tissues and they can be malignant (cancerous) or benign (non-cancerous). Internal pressure and compromised functioning of the brain are the results of the slowly growing but less aggressive benign tumors [6]. The 5-year relative survival rate of patients with malignant brain tumors, however, is only 36% [7]. For example, 2019 has seen 347,992 new cases of brain cancer with 187,491 (54%) males and 160,501 (46%) females. According to [8] in 2019, 138,605 male and 107,648 female deaths attributed to brain cancer were recorded worldwide.

Secondary or metastatic brain tumors usually spread from other areas such as lungs, breasts, skin, colon, etc., towards the brain [2]. The secondary tumors are composed of the same type of cells as the primary tumors.

**Figure 1:**
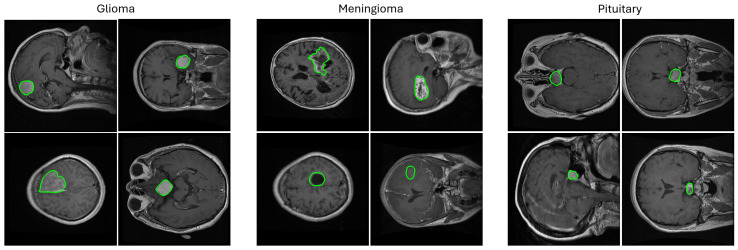
Random samples from the 2D T1-weighted CE-MRI dataset [10] used in this study showing images containing three types of brain tumors (meningioma, glioma, and pituitary) in axial, coronal, and sagittal views with ground truth segmentation masks overlaid. The challenges associated with this and other similar brain tumor datasets are the varying tumor sizes, intensities, and textures that range from large bright areas with high contrast surroundings to small dark areas with barely perceivable outlines. The segmentation masks are shown in green contours.

The main categories of brain tumors are glioma, meningioma, and pituitary tumors as shown in Figure 1. Glioma originates in the glial cells and is associated with a higher mortality degree [2]. Low-grade glioma (LGG) and high-grade glioma (HGG) are the two forms of glioma where LGG is less lethal and has generally a better outcome than HGG. Meningioma originates in the meninges—the outer three tissue layers that separate the brain and the skull  [9]. Pituitary tumors are abnormal cellular cysts in the pituitary gland, which produces the endocrine hormones. Most pituitary tumors are not cancerous [9].

Surgery, chemotherapy, and radiotherapy are often required to treat brain tumors. Timely diagnosis of brain pathology is crucial for the patient’s survival. Magnetic Resonance Imaging (MRI) is the standard for radiation-free diagnostic neuroimaging with an excellent soft tissue contrast, high resolution, and multiplanar capability. Thus, the evaluation of soft tissue tumors is now predominantly performed through the MRI, which has replaced computed tomography (CT). MRI brain scans are first used to classify images into cancerous (tumor) and normal (no tumor). Then, further classification of tumors is needed as outlined above. However, tumors often have hazy boundaries and diverse morphology presenting significant visual recognition challenges for manual MRI image analysis by radiologists. Additionally, there is the problem of background noise and low contrast in MRI images [11]. Finally, the manual assessment of radiographs is time-consuming, non-reproducible, and even subjective in some cases.

AI and deep learning can greatly assist in brain tumor classification and triage solutions to detect benign and malignant cases. In practice, clinicians rely on real-time data to make decisions given the life-changing consequences for patients. Thus, edge devices that can provide close to real-time analysis results are preferred. This requires CPU- and GPU-enabled computational platforms with embedded machine and deep learning algorithms, often referred to as medical edge computing. There are several advantages associated with medical edge computing, such as enhanced patient care, low latency for critical applications, effective use of computational resources, scalability, and flexibility. Additionally, instead of sending images to centralized servers for analysis in medical diagnostics, data can be processed locally on the edge device, thus accelerating diagnosis and treatment.

Accurate brain tumor classification from MRI images using deep learning has recently received great attention in the literature [2,5,11,12,13,14,15]. However, there are comparably fewer works that consider medical edge computing applied to brain tumor classification [3,16,17,18] and that explore the complexity and efficiency of the deep learning models.

Our main contributions can be summarized as follows:

- We propose a shallow convolutional neural network (CNN) architectural framework for brain tumor classification in MRI data. This architecture includes spatial and channel attention ensembling and is particularly suited for medical edge computing.
- We investigate several state-of-the-art model compression techniques which substantially reduce model complexity and training and inference times.
- We quantify the tradeoff between the drop in accuracy and the gain in inference time.
- To test the generality of our model, we perform validation on three different public brain tumor MRI datasets.
- Our code is made publicly available at https://github.com/nirajbabar/Edge-Computing-for-Brain-Tumor-Classification (accessed on 1 June 2024).

This paper is organized as follows. Section 1 provides an overview of the most recent work on brain tumor classification in MRI data. Our methodology in Section 2 outlines the proposed architecture, datasets used, experimental setup, and implementation details. The experimental Section 3 describes the experiments on the three datasets used. We finish with the Discussion in Section 4 and the Conclusion in Section 5.

Recent works on brain tumor classification in MRI have predominantly focused on improving model performance by leveraging advanced deep learning techniques. As seen in Table 1, the research from 2024 and 2025 can be broadly categorized into several key approaches.

A significant portion of the work utilizes transfer learning from pre-trained models. This approach, as demonstrated by Agrawal et al. [19] and Khaliki et al. [14], leverages the feature-extraction capabilities of large-scale models such as InceptionV3 and VGG16 to achieve high accuracies (e.g., 99%). Similarly, Mathivanan et al. [20], Shoaib et al. [21], and Bibi et al. [22] all employed various pre-trained backbones (e.g., DenseNet, MobileNet, and EfficientNet) to fine-tune their models for tumor classification.

**Table 1:** Summary of the reviewed papers. We report the best performing models clustered into two groups according to the use of datasets and further ranked in descending classification accuracy. The Cheng et al. [10] dataset is also known as the Figshare dataset.

Author	Citation	Year	Dataset	Model	Accuracy	Precision	Recall	F1 Score
Ullah et al.	[23]	2024	Cheng et al. [10]	InceptionResnetV2	99.80%	-	-	-
Pacal et al.	[24]	2025	Cheng et al. [10]	NeXtBrain	99.78%	99.69%	99.84%	99.77%
Mathivanan et al.	[20]	2024	Cheng et al. [10]	MobileNetv3	99.61%	98.28%	99.75%	99.00%
Agrawal et al.	[19]	2024	Cheng et al. [10]	InceptionV3	99.57%	99.56%	99.46%	99.50%
Haque et al.	[25]	2024	Cheng et al. [10]	NeuroNet19	99.31%	99.27%	99.27%	99.27%
Khaniki et al.	[26]	2024	Cheng et al. [10]	Cross ViT	99.24%	99.20%	99.27%	99.23%
Kadhim et al.	[27]	2024	Cheng et al. [10]	ResNet50	98.85%	-	-	98.10%
Shaik et al.	[28]	2024	Cheng et al. [10]	MedTransNet	98.37%	98.18%	98.27%	98.16%
Dutta et al.	[29]	2024	Cheng et al. [10]	GT-Net	97.11%	-	-	96.39%
Dutta et al.	[30]	2024	Cheng et al. [10]	ARM-Net	96.64%	96.46%	96.09%	96.20%
Mohanty et al.	[31]	2024	Cheng et al. [10]	Dolphin-SCA-based	95.10%	95.11%	95.06%	95.14%
Hekmat et al.	[32]	2025	Cheng et al. [10]	CLAHE + DWT	94.28%	94.33%	92.33%	93.00%
Shoaib et al.	[21]	2024	Nickparvar [33]	DenseNet201	100%	100%	100%	100%
Ullah et al.	[34]	2024	BraTS2020-21 [35,36]	ResNet-50	99.80%	-	-	-
Ishfaq et al.	[37]	2025	Kaggle [33,38,39]	Custom CNN	99.751%	98.67%	-	98.65%
Rastogi et al.	[40]	2024	Br35H [41]	Multi-branch Net	99.30%	-	-	95.64%
Asiri et al.	[42]	2024	Pashaei et al. [43]	ICA Model	98.90%	-	-	-
Bibi et al.	[22]	2024	SARTAJ [38] and Br35H [41]	InceptionV4	98.70%	99.00%	98.20%	99.10%
Krishnan et al.	[44]	2024	Nickparvar [33]	RViT	98.60%	98.40%	98.75%	98.60%
Khaliki et al.	[14]	2024	Brain Tumor Dataset [38]	VGG16	98.00%	98.00%	98.00%	97.00%
Remzan et al.	[45]	2024	Nickparvar [33]	MLP Ensemble	97.71%	97.71%	97.71%	97.70%
Oztel	[46]	2025	Bhuvaji [38]	Ensemble CNNs-ViT	84.35%	87.32%	85.28%	84.06%

Another major trend is the integration of attention mechanisms and Transformers. These models are designed to improve feature selection by focusing on informative regions. Dutta et al. [30] incorporated spatial attention to enhance their ARM-Net’s feature detection, while Dutta et al. [29] used a Global Transformer Module (GTM) to better select features across different dimensions. More advanced Vision Transformer (ViT) architectures have also been applied, with Khaniki et al. [26] introducing a selective cross-attention mechanism and Krishnan et al. [44] developing a Rotation-Invariant ViT (RViT) to handle different image orientations.

Hybrid and ensemble models are also popular as they combine the strengths of multiple architectures. For example, Remzan et al. [45] used both feature and stacking ensembles of ResNet-50 and DenseNet-121, achieving high AUC and accuracy scores. A recent work by Pacal et al. [24] further exemplifies this by combining CNN and Transformer architectures to create a powerful hybrid model named NeXtBrain. Additionally, some authors [21,42] combined deep learning feature extraction with classical machine learning methods like SVM for the final classification.

Beyond architectural design, other works focused on optimization and data preprocessing. This includes implementing custom, often lightweight, CNNs [19,31] and using optimization algorithms. For instance, Kadhim et al. [27] used Particle Swarm Optimization (PSO) to improve feature selection, while Ullah et al. [23,34] used sparse autoencoders to handle imbalanced datasets and various evolutionary algorithms for hyperparameter tuning. Other preprocessing techniques, such as adaptive filtering for noise reduction [42] and histogram equalization for contrast enhancement [32], were also explored to improve model performance.

Most recent 2025 studies on brain tumor classification from MRI data are converging on hybrid, multi-faceted approaches to achieve superior performance. Ishfaq et al. [37] and Ismail Oztel [46] both employ transfer learning. While Ishfaq et al. use a custom CNN focused on computational efficiency for a ten-class prediction system, Ismail Oztel enhance their approach with image preprocessing using wavelet transforms to capture more detailed features and then use an ensemble of top-performing models. Hekmat et al. [32] also utilize image preprocessing with CLAHE and DWT for feature enhancement before a feature fusion architecture using DenseNet models. The most advanced method, NeXtBrain by Pacal et al. [24], represents a sophisticated hybrid architecture that combines a specialized convolutional block to capture local details and a Transformer block to model global spatial relationships achieving remarkable 99.78% classification accuracy.

As is clear from the above, while there has been a large number of recent works on designing accurate classification models, there is in contrast no attempts to date to investigate and to quantify the effects of model compression on accuracy and inference speed for brain tumor classification in MRI data. This leaves a significant gap in the literature.

## 2. Materials and Methods

### 2.1. Proposed Baseline Model

We build our classification baseline upon the architecture first developed in our previous work in [3]. However, we do not employ the residual skip connections (as they have been shown to contribute little to the overall accuracy) and extend the network in [3] by an additional convolutional block.

**Figure 2:**
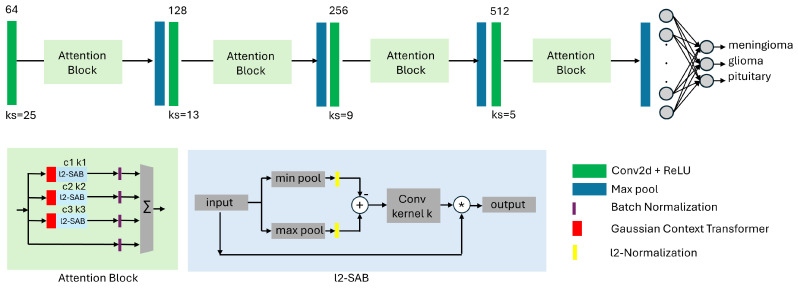
Proposed shallow attention-based CNN architecture for classification of brain tumors in MRI data. The model contains spatial awareness (l2-SAB) blocks from our previous work in [3] and channel attention GCT (Gaussian Context Transformer) blocks [47]. The convolutional backbone consists of only four convolutional layers with increasing filter size and decreasing kernel size, followed by max pooling layers. The difference compared to [3] is that we stack l2-SAB and GCT blocks with varying parameters to increase the accuracy of the network. We term this configuration ANSA_Ensemble.

Therefore, the convolutional backbone of our baseline model consists of only four convolutional layers followed by max pooling layers. We specifically do not employ transfer learning with large pre-trained models like VGG16 or ResNet because of their large number of parameters and their memory footprint. The overview of our architecture is provided in Figure 2. The novelty of our design approach is that we stack a number of l2-SAB [3] and GCT (Gaussian Context Transformer) [47] blocks to create an attention-based (channel and spatial) shallow classification model, which we term ANSA_Ensemble. We specifically select the GCT for our channel attention as this mechanism is parameter-free without additional computational overhead.

For the design of the spatial attention block l2-SAB, we adapt the notations from [48]. Given a spatial attention map $M_{s}\;\in \;{I\;R}^{1\;\times \;H\;\times \;W}$ and an intermediate feature map $F\;\in \;{I\;R}^{C\;\times \;H\;\times \;W}$, the output $F^{\prime }$ of the l2-SAB can be written as follows (applied to each channel separately):

(1) $$F^{\prime }\;=\;M_{s}\;(\;F\;)\;\;⊗\;F$$

where ⊗ denotes element-wise multiplication. Further, we use two pooling operations, $F_{max}^{s}\;\in \;{I\;R}^{1\;\times \;H\;\times \;W}$ and $F_{min}^{s}\;\in \;{I\;R}^{1\;\times \;H\;\times \;W}$. These operations produce two 2D feature maps that are further $l_{2}$-normalized, subtracted from each other, and convolved by a convolutional layer with kernel size *K*:

(2) $$M_{s}\left(F\right)\;=\;\sigma \left(f^{K\;\times \;K}\left(l_{2}\left(MaxPool\left(F\right)\right)\;-\;l_{2}\left(MinPool\left(F\right)\right)\right)\right)$$

where $f^{K\;\times \;K}$ is a convolution operation with the filter size K × K and σ denotes the sigmoid activation function.

Each l2-SAB block has a hyperparameter *k* denoting the kernel size of the corresponding convolution operations (see Figure 2) while each GCT block has a hyperparameter *c* that controls the spread of the Gaussian function.

### 2.2. Model Compression Methods

The aim of model compression methods is to increase the model’s efficiency by reducing its size and memory footprint, thus making it suitable for edge computing. The most common types of model compression methods include pruning, low-rank factorization, knowledge distillation, and transfer learning [49]. In this work we focus on the following approaches:

#### 2.2.1. Depthwise-Separable Convolutions (DSCs)

DSC [50] aims to reduce a model’s overall parameters and computational cost, ideally with a minimal tradeoff in accuracy. It splits the typical convolution step into two, such that the first (depthwise) step captures each channel of the input image individually, while the second (pointwise) step aggregates information from all input channels.

#### 2.2.2. Reduced Feature Maps (RFMs)

In order to reduce the complexity of a model, the simplest approach is to reduce the number of feature maps. In this configuration all models have a reduced number of channels in the convolutional layers. We reduce the number of filters from 64-128-256-512 to 8-16-32-64 in corresponding convolutional layers.

#### 2.2.3. Parameter-Efficient Fine-Tuning (PEFT)

LoRA [51] is a recent type of a parameter-efficient fine-tuning (PEFT) method that decomposes weight matrices into low-rank components. This model compression approach significantly reduces the number of trainable parameters, which speeds up fine-tuning and results in a smaller memory footprint. The PEFT library from Huggingface enables the parameter efficient adaptation of pre-trained language models for various downstream applications without fine-tuning all of the model’s parameters. This library supports multiple fine-tuning methods such as LoRA, which is not restricted to prompt-like Transformer architectures and can be applied to, e.g., Multi-Layer Perceptrons (MLPs) (https://huggingface.co/docs/peft/v0.8.0/en/developer_guides/custom_models accessed on 1 June 2024). In our architecture we apply LoRA to all convolutional and all fully connected layers in our models.

#### 2.2.4. Canonical Polyadic Tensor Decomposition (CPTD)

There are many tensor decomposition methods such as Kroenecker, Tucker, Canonical Polyadic, SVD, etc., but the availability of code to implement these methods is limited. Therefore, for practicality reasons, we focus on the Canonical Polyadic Tensor Decomposition [52] (CPTD) that takes the current convolution layer and breaks it down into small and quickly executable sequences of layers that utilize the weights of the inputted tensor to conduct the tensor calculations. Using the weights lowers the complexity of the convolutional layers and is used in hopes of reducing the model parameters of all the machine learning models. This approach is similar to MobileNets; however, this form of tensor decomposition allows for more separability in the spatial dimensions. Furthermore, during the implementation of this approach, it is suggested to use a lower learning rate to prevent any errors in the training process.

**Figure 3:**
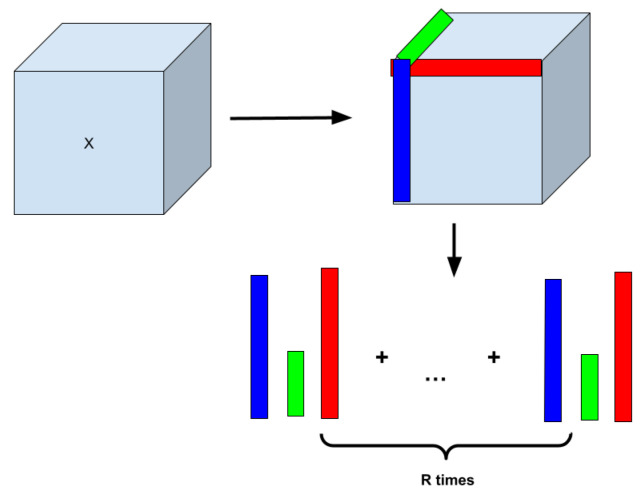
Concept of Canonical Polyadic Tensor Decomposition (CPTD) that factorizes a tensor into a sum of outer products of vectors.

Figure 3 shows an illustrative example of how Canonical Polyadic Tensor Decomposition [53] works. The algorithm takes a certain range of rows of each tensor dimension to calculate the tensor’s outer product and the weighted tensor. From there, each piece of data is optimized so that the upcoming layers can properly use the data without any errors in the data. The equation for the calculations is as follows: $\underset{\hat{X}}{min}||X\;-\;\hat{X}\left|\right|$, where *X* is the original tensor and $\hat{X}\;=\;\sum_{r\;=\;1}^{R}x_{ri}y_{rj}z_{rk}$ (*R* is the number of rows and $\hat{X}$ is the tensor decomposed tensor) to conduct calculations for [53].

Because of the high computational burden, we decompose only the first convolutional layer in all three models, AlexNet, ANSA, and ANSA_Ensemble.

#### 2.2.5. Pruning (PR)

Pruning is a technique used to reduce the complexity and size of a model by removing the less significant neurons which are chosen based on several criteria. The process of pruning aims to enhance the efficiency of the model, providing faster inference times and lower memory usage, which are helpful for deployments on low-resource devices. It also helps in preventing overfitting (if performed carefully) by simplifying the model to avoid a significant loss in accuracy. This method is commonly employed in complex CNNs. There are several methods for pruning neural networks [54] from which we choose the magnitude-based pruning to optimize the proposed neural network.

The idea of pruning networks by removing weights based on their saliency was first introduced by LeCun et al. [55] in 1989, which was improved over the years in [56,57]. Song Han et al. [58] showcased how magnitude-based pruning could reduce the size of models like AlexNet and VGG-16 by up to 35× and 49×, respectively, without losing accuracy.

### 2.3. Datasets

#### 2.3.1. Dataset 1

The Cheng et al. [10] dataset (https://figshare.com/articles/dataset/brain_tumor_dataset/1512427 accessed on 1 June 2024) contains 3064 2D T1-weighted contrast-enhanced MRI image scans. This dataset was collected from 2005 to 2010 from Nanfang Hospital in Guangzhou, China, and General Hospital at Tianjing Medical University, China. Each slice information is provided in a .mat (MATLAB) format, containing the patient ID, image array, classification label, tumor border array, and tumor mask. The dataset was collected from 233 patients and the labels consist of three types of primary brain tumors, such as meningioma (708 slices), glioma (1426 slices), and pituitary tumor (930 slices). Samples are collected in axial, coronal, and sagittal orientations. Most images have a resolution of 512 × 512 pixels, but there are also some images of 256 × 256 pixel resolution.

#### 2.3.2. Dataset 2

The Bhuvaji et al. [38] dataset contains 3264 T1, T2, and FLAIR brain MRI image scans split into the following categories: glioma, meningioma, pituitary, and no tumor. The dataset contains 926 glioma tumor MRIs, 937 meningioma tumor MRIs, 500 no tumor MRIs, and 901 pituitary tumor MRIs. All images have a .jpg file extension and most images have a resolution of 512 × 512 pixels. This resolution did not match the resolution accepted by our model so we needed to resize each image to fit the 256 × 256 pixel resolution our model accepted. Images were added to a master folder, resized to 256 × 256 pixel resolution, and then shuffled into training, validation, and testing sets equally based on the picture training splits. The summary statistics of all datasets is given in Table 2.

#### 2.3.3. Dataset 3

The Sherif et al. [59] dataset contains 4292 MRI images, split into four categories: glioma, meningioma, pituitary, and no tumor (normal brain tissue). The dataset has 1038 glioma, 1318 meningioma, 1255 pituitary, and 681 no tumor MRI images. The vast majority of images have a .jpg file extension; however, a select few were either .png or .jpeg and were converted to .jpg in the data preparation process. Most files had the 512 × 512 image resolution. Therefore, we applied resizing to 256 × 256. As the dataset is initially organized into its own testing and training sub-directories, we combined them in a manner that resulted in one master directory, which contained four sub-directories (each about one category), with the images for that sub-category within each sub-directory. We only performed image resizing, image type conversion, and shuffling into training, validation, and testing sets after this step.

All three datasets are large, imbalanced, and challenging due to variability in tumor image characteristics, shapes, and sizes. We performed a random 70%-10%-20% split to obtain images for training, validation, and test.

### 2.4. Implementation Details

The processing pipeline was implemented in Python 3.9 and the deep learning open-source library PyTorch 2.0.1. All experiments were performed on a desktop computer with the Ubuntu operating system 18.04.3 LTS with the Intel(R) Core(TM) i9-9900K CPU, Nvidia GeForce RTX 2080 Ti GPU, and a total of 62GB RAM.

We trained all models using an Adam optimizer [60] with a learning rate of lr = 0.00001. The batch size was 64 and all models were trained for 200 epochs with an early stopping with patience = 20 epochs. All images were resized to 256×256 as an input to all models. We applied data augmentation but did not preprocess the input images otherwise.

### 2.5. Data Augmentation

The datset was expanded and diversified by implementing seven different transformations to each image as shown in Table 3. Each transformation was applied to the images with an equal probability, resulting in a doubling of the image count across all three datasets. We used the albumentations library to implement the proposed data augmentation techniques.

For example, Dataset 1 has 6128 images after our data augmentation step. Dataset 2 has 6528 images and Dataset 3 has 8584 images.

## 3. Results

The comparison with the state-of-the-art for Dataset 1 is provided in Table 4. We note that we train the models ten times in all our experiments and report the best results (corresponding to the highest classification accuracy) in Table 4 as every other model has also reported their best results. Table 5 presents a performance comparison involving state-of-the-art models, all of which report results obtained using five-fold cross-validation methodology.

Multiple metrics are utilized to assess the effectiveness of classification models, including accuracy, precision, recall/sensitivity, F1 score, and specificity. These metrics are defined in Equations (3) through (7):

(3) $$\mathrm{Accuracy}\;=\;\frac{\mathrm{Number}\;\mathrm{of}\;\mathrm{correct}\;\mathrm{predictions}}{\mathrm{Total}\;\mathrm{number}\;\mathrm{of}\;\mathrm{predictions}}$$

(4) $$\mathrm{Precision}\;=\;\frac{\mathrm{True}\;\mathrm{Positives}}{\mathrm{True}\;\mathrm{Positives}\;\;+\;\;\mathrm{False}\;\mathrm{Positives}}$$

(5) $$\mathrm{Recall}/\mathrm{Sensitivity}\;=\;\frac{\mathrm{True}\;\mathrm{Positives}}{\mathrm{True}\;\mathrm{Positives}\;\;+\;\;\mathrm{False}\;\mathrm{Negatives}}$$

(6) $$\mathrm{F1-score}=\frac{2\;\times \;\mathrm{Precision}\;\times \;\mathrm{Recall}}{\mathrm{Precision}\;+\;\mathrm{Recall}}$$

(7) $$\mathrm{Specificity}\;=\;\frac{\mathrm{True}\;\mathrm{Negatives}}{\mathrm{True}\;\mathrm{Negatives}\;\;+\;\;\mathrm{False}\;\mathrm{Positives}}$$

### 3.1. Tradeoff Factor

To evaluate and compare different model compression techniques, we define a tradeoff factor θ as follows:

(8) $$\theta \;=\;\left(Acc_{b}\;-\;Acc_{m}\right)\;-\;\left(t_{b}-t_{m}\right)$$

where $Acc_{b}$ and $Acc_{m}$ are classification accuracy in % on the test set for the baseline and each compressed model, respectively. In milliseconds, $t_{b}$ and $t_{m}$ are the average inference time per image (computed on GPU). A lower θ indicates a better tradeoff between the classification accuracy and speed.

We show the quantitative evaluation of the tradeoff factors for all three datasets in Table 6, Table 7 and Table 8. Here, we reproduce the AlexNet and ANSA models from our previous work [3].

The AlexNet model is a shallow version of [64] with only three convolutional and max pooling layers. While the number of filters increases in this model with the depth of the network, the convolutional kernel sizes decrease with the depth of the network.

The ANSA (AlexNet Spatial Awareness) model is an attention-guided and l2-normalized AlexNet with added l2-SAB attention blocks. This is the model developed in our previous work in [3].

The ANSA_Ensemble models used in our experiments have *k* and *c* hyperparameters as shown in Table 9.

In magnitude-based pruning we optimize the model by removing weights based on their magnitudes. To implement this we train the model until it reaches an acceptable performance level. Once the model is trained, the weights are ranked according to their absolute values. The weights having smaller magnitudes are considered less important to the model’s performance. A certain percentage of these smallest weights is then pruned by masking them to zero. This reduction simplifies the model and results in decreased memory usage. This initially reduces the model’s accuracy so it is fine-tuned again making sure that the pruned weights remain zero and the unpruned weights are updated to recover performance loss.

**Figure 4:**
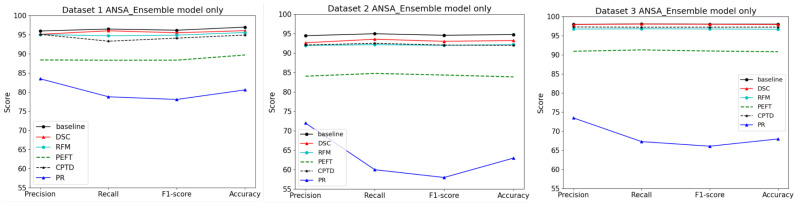
Performance of the ANSA Ensemble model on three datasets using different model compression methods. It can be seen that pruning (PR) has the worst performance while DSC (depthwise-separable convolutions) has the best performance compared to the baseline.

Performance plots in Figure 4 show that the best performance is achieved with DSC while PR is the worst performing model compression technique. Table 6, Table 7 and Table 8 also verify that the best tradeoff factor (lowest negative values) corresponds to the DSC and RFM techniques for all three datasets. That means DSC and RFM result in the smallest accuracy drop and highest gain in inference speed when compared against other methods.

**Figure 5:**
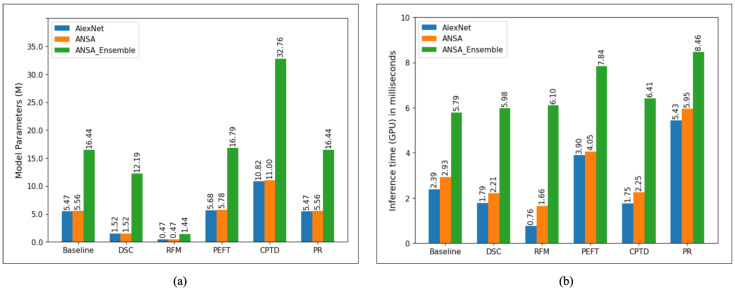
(**a**) Total number of model parameters (in millions) for all model compression methods investigated. It can be seen that RFM has the lowest numbers for all models while our implementation of CPTD has the highest. (**b**) Inference time on the GPU (in milliseconds) per image computed on the test set.

We also visualize the total number of model parameters and the inference time per image (on GPU in milliseconds) in Figure 5, where it can be seen that model complexity is greatly reduced by RFM, followed by DSC. This finding correlates with the GPU inference time, which is also shown in Figure 5.

### 3.2. Monte Carlo Simulations with a 70-10-20 Data Split

We perform Monte Carlo simulations for the ANSA Ensemble and ANSA Ensemble with DSC models on each of the three datasets. Each model is trained 10 times, and the average performance metrics along with the standard deviation are reported. In these simulations, 70% of the data is used for training, 10% for validation, and 20% for testing. Table 10, Table 11 and Table 12 present the performance metrics for all three datasets.

### 3.3. Monte Carlo Simulations with Five-Fold Cross Validation

We perform Monte Carlo simulations with five-fold cross-validation for the ANSA Ensemble and ANSA Ensemble with DSC models on each of the three datasets. This method divides the dataset into five equal parts, iteratively training on four parts and testing on the remaining part. The average performance metrics along with the standard deviation are reported in Table 13, Table 14 and Table 15.

### 3.4. Grad-CAM Simulations

**Figure 6:**
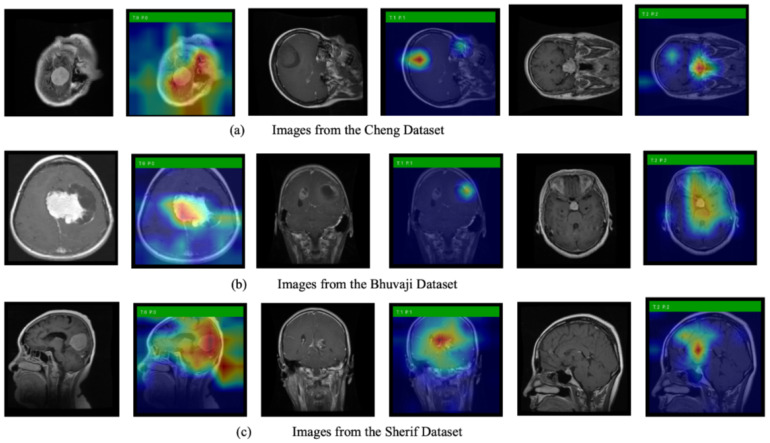
Original MRI images along with images overlayed with the heatmap generated using Grad-CAM.

We also use Grad-CAM, an explainable AI technique, to show the model’s decision-making process. Figure 6 displays the original images alongside heatmaps overlaid on them. The regions shown in red represent the primary focus of the model. The first row of images is from the Cheng dataset, the second row from the Bhuvaji dataset, and the third row from the Sherif dataset. It is clear that in all cases, the models prominently focus on the tumor region, which enhances the trustworthiness of the model’s decisions.

### 3.5. Cross-Dataset Generalization

This subsection discusses cross-dataset generalization. Table 16 shows the results of models trained on the Cheng dataset but tested on the Sherif and Bhuvaji datasets. The Cheng dataset contains three classes—glioma, meningioma, and pituitary—while the Bhuvaji and Sherif datasets include an additional class, no tumor. To address this difference, we fine-tune the last layer of the model trained on the Cheng dataset separately for the Bhuvaji and Sherif datasets to accommodate the fourth class. We then perform predictions and the results reported. Table 17 presents the results of models trained on the Bhuvaji dataset and tested on the Cheng and Sherif datasets. The model is fine-tuned prior to testing on the Cheng dataset but not before testing on the Sherif dataset, as both share the same number of classes. Table 18 shows the results of the models trained on the Sherif dataset and tested on the remaining two datasets.

### 3.6. Ablation Study

We conducted an ablation study to evaluate the effectiveness of the proposed channel and spatial attention blocks. Table 19, Table 20 and Table 21 present the results of experiments performed on all three datasets. The ANSA Ensemble includes the proposed channel attention block four times. For the ablation study, we measure performance by removing one attention block at a time. Since the channel attention block also contains Gaussian Context Transformer (GCT), we assess the impact of GCT by training models without it as well.

All three tables demonstrate that increasing the number of attention blocks along with GCT improves performance, indicating that the presence of attention blocks enhances the model’s effectiveness.

## 4. Discussion

The results across all experiments highlight several important findings regarding the performance of the proposed ANSA Ensemble and its compressed variants, especially when compared to state-of-the-art models. In Dataset 1, the ANSA Ensemble achieved competitive accuracy, only slightly lower than the best CNN Ensemble model, while maintaining significantly reduced inference times compared to large architectures such as fine-tuned VGG16. This shows the efficiency of the model’s attention-guided design, which balances accuracy and speed effectively.

The five-fold cross-validation experiments further confirm the robustness of the ANSA Ensemble architecture, showing stable performance with low variance in precision, recall, F1 score, and specificity. The DSC-compressed models showed slight drops in accuracy, but they consistently reduced inference time and model complexity, demonstrating the effectiveness of depthwise-separable convolutions in achieving better speed–accuracy tradeoffs compared to pruning, which consistently yielded the poorest results both in accuracy retention and tradeoff factor.

The tradeoff factor analysis across all three datasets provides quantitative support for these observations. The DSC and RFM methods consistently delivered the smallest accuracy drop along with notable speed gains, offering the most balanced compromise between performance and efficiency. By contrast, pruning substantially degraded accuracy, suggesting that aggressive weight removal may disproportionately affect feature extraction.

Monte Carlo simulation results with both 70-10-20 data splits and five-fold cross-validation further highlight the stability of the ANSA Ensemble architecture, with standard deviations remaining low across runs. Despite the slight accuracy reductions observed in models compressed using DSC, the improvements in computational efficiency and reduced parameter counts are likely beneficial in real-world settings where inference speed and deployment feasibility are critical, such as in point-of-care systems.

Explainability analyses using Grad-CAM revealed that both the baseline and compressed models consistently focused on tumor regions across the Cheng, Bhuvaji, and Sherif datasets. This alignment between model attention and clinically relevant regions enhances trust in the decision-making process and underscores the suitability of ANSA-based architectures for medical applications.

Cross-dataset generalization experiments showed a notable performance drop when models were tested on datasets with different class distributions, particularly when moving from a three-class to a four-class problem. This suggests that while the core features learned by the ANSA Ensemble are transferable, fine-tuning the final classification layers remains necessary to account for domain and label distribution shifts.

The ablation study provides strong evidence of the importance of attention blocks and the integration of Gaussian Context Transformer (GCT) modules. Incrementally adding attention blocks consistently improved accuracy, precision, recall, and F1 score across all datasets. Furthermore, the presence of GCT consistently boosted performance over configurations without it, indicating that the spatial and channel attention mechanisms contribute significantly to the ability of the model to capture clinically relevant features in MRI brain scans.

Overall, the proposed ANSA Ensemble demonstrates a compelling balance between accuracy, efficiency, and interpretability. While the highest absolute accuracy was achieved by larger ensemble CNN models, the ANSA Ensemble model with its integrated spatial awareness offers a practical alternative for real-world clinical deployment.

Although the proposed model demonstrates promising performance, its accuracy is likely to improve further with an increase in the number of attention blocks and the overall depth of the network. However, deeper architectures inherently introduce a larger number of trainable parameters, which leads to increased computational cost and memory consumption. To address this tradeoff between model complexity and efficiency, future work will explore advanced model compression and optimization strategies.

In particular, we plan to investigate the Lottery Ticket Hypothesis [65]-based model compression technique. This approach suggests that dense neural networks contain smaller subnetworks, which are also known as winning tickets, which can match the performance of the full model when trained independently. This idea enables the extraction of sparse, efficient architectures that retain accuracy while substantially lowering parameter count and computational cost. Such models offer a promising path toward lightweight, high-performing systems suitable for deployment in resource-constrained environments.

## 5. Conclusions

This work presents the ANSA Ensemble, an attention-guided deep learning model designed for brain tumor classification from MRI images. The key novelty of the proposed method lies in its integration of spatial awareness through l2-normalized attention blocks combined with Gaussian Context Transformers (GCTs), which together enable precise and robust focus on tumor regions. This architecture outperforms several existing models by effectively capturing both spatial and channel-wise contextual information, resulting in improved classification accuracy and interpretability.

The ANSA Ensemble achieves competitive accuracy compared to state-of-the-art CNN ensembles while offering significant gains in computational efficiency and reduced inference time. Among model compression techniques, depthwise-separable convolutions (DSCs) prove most effective at maintaining accuracy with improved speed and lower parameter counts, highlighting the potential for deploying lightweight yet powerful models in clinical practice.

Comprehensive evaluations using multiple datasets, various validation methods, and Monte Carlo simulations demonstrate the robustness and stability of the proposed models. The explainability analyses using Grad-CAM further reinforce the clinical validity of the model by showing its attention aligns with medically relevant tumor regions. Ablation studies confirm the added value of each attention block and GCT component in boosting classification performance.

Overall, the ANSA Ensemble model offers a unique combination of accuracy, interpretability, and efficiency, making it a promising candidate for real-world clinical applications in brain tumor diagnosis. Its novel attention mechanisms and effective compression strategies provide tangible value in enhancing diagnostic precision while enabling practical deployment on resource-constrained systems. 

## Figures and Tables

**Table 2:** Summary of open-source datasets used.

Features	Dataset 1	Dataset 2	Dataset 3
Citation	Cheng et al. [10]	Bhuvaji et al. [38]	Sherif et al. [59]
Number images	3064	3264	4292
Modality	T1w CE-MRI	T1, T2, FLAIR	MRI
Classes	3	4	4
	Glioma: 1426	Glioma: 926	Glioma: 1038
	Meningioma: 708	Meningioma: 937	Meningioma: 1318
	Pituitary: 930	Pituitary: 901	Pituitary: 1255
		No tumor: 500	No tumor: 681
Input resolution	512 × 512	512 × 512	512 × 512

**Table 3:** Data augmentation details.

Transformation	Value
Vertical Flip	True
Horizontal Flip	True
Rotation	90
Width Shift Range	0.1
Height Shift Range	0.1
Zoom Range	0.2
Shear Range	0.2

**Table 4:** Quantitative evaluation on the Cheng et al. Dataset 1 and comparison with the state of the art. Our baseline model ANSA_Ensemble has a slightly lower accuracy than the best performing CNN Ensemble model.

Classification Model	Year	Result from:	Accuracy	Evaluation	# Parameters	Inf. Time (CPU)
BoW+classifier [10]	2015	Original paper	91.28%	Not Available	Not Available	Not Available
CNN [61]	2020	Reproduced	95.27%	70%-10%-20%	1,282,955	10 ms
CNN [62] Transfer Learning	2022	Original paper	95.75%	80%-20%	-	-
CNN Ensemble [7]	2022	Original paper	98.67%	70%-30%	-	-
AlexNet [3]	2023	Reproduced	94.78%	70%-10%-20%	4,003,011	16 ms
AlexNet + CBAM [48]	2023	Reproduced	95.60%	70%-10%-20%	5,474,547	20 ms
Fine-tuned VGG16 [3]	2023	Reproduced	93.31%	70%-10%-20%	114,239,171	109 ms
ANSA [3]	2023	Reproduced	96.57%	70%-10%-20%	7,293,523	19 ms
ANSA + VGG16 ensemble [3]	2023	Reproduced	96.74%	70%-10%-20%	15,516,179	115 ms
MobileNetv3 [20]	2024	Original paper	99.61%	80%-20%	-	-
NeXTBrain [24]	2025	Original paper	99.78%	70%-15%-15%	∼23,910,000	-
ANSA_Ensemble (ours)		Implemented	98.04%	70%-10%-20%	16,442,470	68 ms
ANSA_Ensemble + DSC (ours)		Implemented	96.43%	70%-10%-20%	12,190,884	58 ms

**Table 5:** Comparative analysis of methods based on five-fold cross-validation results.

Classification Model	Year	Result from:	Accuracy	Evaluation	# Parameters	Inf. Time (CPU)
CNN [63] Transfer Learning	2019	Original paper	93.0%	five-fold	-	-
InceptionV3-based [19]	2024	Original paper	99.57%	five-fold	22,039,075	-
ARM-Net [30]	2024	Original paper	96.64%	five-fold	1,133,000	-
ANSA_Ensemble (ours)		Implemented	96.69%	five-fold	16,442,470	68 ms
ANSA_Ensemble + DSC (ours)		Implemented	95.87%	five-fold	12,190,884	58 ms

**Table 6:** Tradeoff factors for the Cheng dataset.

Model	$\theta_{\mathrm{PEFT}}↓$	$\theta_{\mathrm{DSC}}↓$	$\theta_{\mathrm{RFM}}↓$	$\theta_{\mathrm{CPTD}}↓$	$\theta_{\mathrm{PR}}↓$	${\mathrm{Acc}}_{b}↑$
AlexNet	6.556	−0.7622	−1.6246	12.412	4.021	94.127
ANSA	6.995	2.218	0.03699	4.215	10.200	96.737
ANSA Ensemble	9.556	1.338	1.947	2.902	19.478	97.227

**Table 7:** Tradeoff factors for the Bhuvaji dataset.

Model	$\theta_{\mathrm{PEFT}}↓$	$\theta_{\mathrm{DSC}}↓$	$\theta_{\mathrm{RFM}}↓$	$\theta_{\mathrm{CPTD}}↓$	$\theta_{\mathrm{PR}}↓$	${\mathrm{Acc}}_{b}↑$
AlexNet	6.905	0.456	0.457	18.096	0.479	88.497
ANSA	7.511	0.918	2.448	4.593	22.424	92.94
ANSA Ensemble	11.045	2.760	1.691	2.909	31.79	94.94

**Table 8:** Tradeoff factors for the Sherif dataset.

Model	$\theta_{\mathrm{PEFT}}↓$	$\theta_{\mathrm{DSC}}↓$	$\theta_{\mathrm{RFM}}↓$	$\theta_{\mathrm{CPTD}}↓$	$\theta_{\mathrm{PR}}↓$	${\mathrm{Acc}}_{b}↑$
AlexNet	6.65	−3.193	−0.539	12.431	3.404	91.51
ANSA	3.609	−1.593	−0.287	0.484	28.714	97.81
ANSA Ensemble	12.322	−0.069	1.202	−4.520	30.403	98.08

**Table 9:** Parameters of l2-SAB spatial awareness blocks used in all experiments on all datasets.

Attention Block	l2-SAB Kernel Size	GCT c Parameter
	**k1-k2-k3**	**c1-c2-c3**
block 1	8-6-4	4-2-1
block 2	8-6-4	4-2-1
block 3	6-4-2	4-2-1
block 4	1-1-1	4-2-1

**Table 10:** Monte Carlo simulations for the Cheng dataset.

Model	Precision	Recall	F1 Score	Specificity	Accuracy
	**(%)**	**(%)**	**(%)**	**(%)**	**(%)**
ANSA Ensemble	95.76 ± 1.15	95.80 ± 1.05	95.81 ± 1.07	98.36 ± 0.56	96.17 ± 1.03
ANSA Ensemble + DSC	94.61 ± 0.89	94.62 ± 0.94	94.59 ± 0.85	97.78 ± 0.63	95.06 ± 0.88

**Table 11:** Monte Carlo simulations for the Bhuvaji dataset.

Model	Precision	Recall	F1 Score	Specificity	Accuracy
	**(%)**	**(%)**	**(%)**	**(%)**	**(%)**
ANSA Ensemble	94.33 ± 0.92	94.48 ± 0.97	94.39 ± 0.93	98.73 ± 0.28	94.33 ± 0.86
ANSA Ensemble + DSC	92.46 ± 0.77	92.95 ±0.64	92.67 ± 0.70	98.13 ± 0.24	92.56 ± 0.73

**Table 12:** Monte Carlo simulations for the Sherif dataset.

Model	Precision	Recall	F1 Score	Specificity	Accuracy
	**(%)**	**(%)**	**(%)**	**(%)**	**(%)**
ANSA Ensemble	95.19 ± 0.47	95.19 ± 0.66	95.17 ± 0.54	99.04 ± 0.18	95.24 ± 0.52
ANSA Ensemble + DSC	94.38 ± 0.80	94.28 ± 0.78	94.31 ± 0.76	98.74 ± 0.25	94.36 ± 0.76

**Table 13:** Five-fold cross-validation for Cheng dataset.

Model	Precision	Recall	F1 Score	Specificity	Accuracy
	**(%)**	**(%)**	**(%)**	**(%)**	**(%)**
ANSA Ensemble	96.44 ± 0.64	96.30 ± 0.77	96.36 ± 0.70	98.50 ± 0.35	96.69 ± 0.64
ANSA Ensemble + DSC	95.62 ± 0.25	95.34 ± 0.16	95.48 ± 0.18	97.82 ± 0.05	95.87 ± 0.13

**Table 14:** Five-fold cross-validation for Bhuvaji dataset.

Model	Precision	Recall	F1 Score	Specificity	Accuracy
	**(%)**	**(%)**	**(%)**	**(%)**	**(%)**
ANSA Ensemble	94.96 ± 0.48	95.38 ± 0.41	95.19 ± 0.34	98.69 ± 0.12	95.16 ± 0.33
ANSA Ensemble + DSC	94.34 ± 0.43	94.47 ± 0.50	94.37 ± 0.44	98.37 ± 0.17	94.31 ± 0.47

**Table 15:** Five-fold cross-validation for Sherif dataset.

Model	Precision	Recall	F1 Score	Specificity	Accuracy
	**(%)**	**(%)**	**(%)**	**(%)**	**(%)**
ANSA Ensemble	95.11 ± 0.28	95.22 ± 0.45	95.15 ± 0.35	98.37 ± 0.14	95.20 ± 0.40
ANSA Ensemble + DSC	94.47 ± 0.10	94.33 ± 0.38	94.39 ± 0.23	98.09 ± 0.09	94.41 ± 0.24

**Table 16:** Performance of the models trained on the Cheng dataset and tested on the Bhuvaji and Sherif datasets.

Dataset	Model	Precision	Recall	F1 Score	Specificity	Accuracy
		**(%)**	**(%)**	**(%)**	**(%)**	**(%)**
Bhuvaji	ANSA Ensemble	46.03	54.01	49.50	86.45	61.20
Bhuvaji	ANSA Ensemble + DSC	46.04	53.68	49.46	86.26	60.90
Sherif	ANSA Ensemble	47.31	56.09	51.30	87.23	63.38
Sherif	ANSA Ensemble + DSC	47.15	55.37	50.79	87.00	62.80

**Table 17:** Performance of the models trained on the Bhuvaji dataset and tested on the Cheng and Sherif datasets.

Dataset	Model	Precision	Recall	F1 Score	Specificity	Accuracy
		**(%)**	**(%)**	**(%)**	**(%)**	**(%)**
Cheng	ANSA Ensemble	81.83	80.44	80.76	90.67	82.47
Cheng	ANSA Ensemble + DSC	75.24	68.11	69.12	84.12	71.96
Sherif	ANSA Ensemble	92.80	93.88	93.26	98.01	93.83
Sherif	ANSA Ensemble + DSC	84.23	85.04	84.47	95.08	85.09

**Table 18:** Performance of the models trained on the Sherif dataset and tested on the Cheng and Bhuvaji datasets.

Dataset	Model	Precision	Recall	F1 Score	Specificity	Accuracy
		**(%)**	**(%)**	**(%)**	**(%)**	**(%)**
Cheng	ANSA Ensemble	84.49	82.58	83.37	91.55	84.75
Cheng	ANSA Ensemble + DSC	75.20	76.04	75.53	88.58	77.75
Bhuvaji	ANSA Ensemble	95.17	95.39	95.26	98.38	95.25
Bhuvaji	ANSA Ensemble + DSC	86.42	87.57	86.78	95.69	86.99

**Table 19:** Ablation study results on the Cheng dataset.

No of Attention Blocks	Spatial Attention Block Without GCT					Spatial Attention Block with GCT				
	**Precision**	**Recall**	**F1 Score**	**Specificity**	**Accuracy**	**Precision**	**Recall**	**F1 Score**	**Specificity**	**Accuracy**
	**(%)**	**(%)**	**(%)**	**(%)**	**(%)**	**(%)**	**(%)**	**(%)**	**(%)**	**(%)**
0	50.73	62.58	55.77	83.69	72.86	50.73	62.58	55.77	83.69	72.86
1	93.37	91.91	92.56	96.31	93.31	90.44	90.34	90.39	95.35	91.11
2	93.98	92.03	92.88	96.37	93.56	93.91	93.84	93.87	97.08	94.37
3	93.71	93.97	93.84	97.11	94.37	94.80	95.28	95.04	97.70	95.43
4	95.05	95.58	95.30	97.84	95.68	97.75	97.83	97.79	99.02	98.04

**Table 20:** Ablation study results on the Bhuvaji dataset.

No of Attention Blocks	Spatial Attention Block Without GCT					Spatial Attention Block with GCT				
	**Precision**	**Recall**	**F1 Score**	**Specificity**	**Accuracy**	**Precision**	**Recall**	**F1 Score**	**Specificity**	**Accuracy**
	**(%)**	**(%)**	**(%)**	**(%)**	**(%)**	**(%)**	**(%)**	**(%)**	**(%)**	**(%)**
0	7.24	25	11.23	74.99	28.99	7.24	25	11.23	74.99	28.99
1	89.65	89.59	89.59	96.52	89.74	86.00	85.53	85.45	95.08	85.53
2	90.81	89.35	89.73	96.47	89.74	92.18	91.64	91.85	97.19	91.81
3	93.02	92.77	92.88	97.54	92.80	93.95	93.65	93.75	97.85	93.72
4	93.35	93.02	93.15	97.67	93.19	94.35	94.53	94.44	89.06	94.33

**Table 21:** Ablation study results on the Sherif dataset.

No of Attention Blocks	Spatial Attention Block Without GCT					Spatial Attention Block with GCT				
	**Precision**	**Recall**	**F1 Score**	**Specificity**	**Accuracy**	**Precision**	**Recall**	**F1 Score**	**Specificity**	**Accuracy**
	**(%)**	**(%)**	**(%)**	**(%)**	**(%)**	**(%)**	**(%)**	**(%)**	**(%)**	**(%)**
0	7.68	25	11.75	74.99	30.73	7.68	25	11.75	74.99	30.73
1	91.61	91.90	91.77	97.21	91.79	92.12	91.40	91.71	97.14	91.73
2	91.61	91.42	91.49	97.16	91.67	93.47	93.63	93.48	97.80	93.53
3	93.48	93.53	93.50	97.65	93.52	94.68	94.62	94.63	98.12	94.52
4	93.96	94.27	94.11	98.06	94.23	95.16	95.42	95.25	98.42	95.34

## Data Availability

The original data presented in the study are openly available in [10,38,59].

## Abbreviations

The following abbreviations are used in this manuscript:

MRI	Magnetic Resonance Imaging
LGG	Low-grade glioma
HGG	High-grade glioma
CT	Computed Tomography
CNN	Convolutional neural network
PSO	Particle Swarm Optimization
GTM	Global Transformer Module
RViT	Rotation-Invariant Vision Transformer
GCT	Gaussian Context Transformer
PCA	Principal Component Analysis
SVM	Support Vector Machine
MLP	Multi-Layer Perceptron
SAB	Spatial attention block
DSC	Depthwise-separable convolution
RFM	Reduced Feature Maps
PEFT	Parameter-Efficient Fine-Tuning
CPTD	Canonical Polyadic Tensor Decomposition
PR	Pruning
ANSA	AlexNet Spatial Awareness
